# A Few Practical Suggestions on the Management of Chronic Ulcers

**Published:** 1866-12

**Authors:** R. Dexter

**Affiliations:** Chicago


ARTICLE XLVIII.

### A FEW PRACTICAL SUGGESTIONS ON THE MANAGEMENT OF CHRONIC ULCERS.

BY R. DEXTER, M.D., of Chicago.

If an ulcer has become chronic, it is owing to a cause or causes that tend to subvert the reparative powers of the system, thus thwarting nature's efforts to heal the breach. These causes may be local or constitutional, or both; in fact, there are few such sores but have a specific constitutional vice that sustains them. The first question then, regarding the pathology of chronic ulcers, is:—What morbid conditions have made them thus, and by what process has this result been accomplished? The next question is:—What are their precise conditions at the present time; and how much the constitutional fault complicates the local disease; and what are their mutual influences upon each other?

An ulcer has become chronic when it has passed through the ordinary acute stages attending such maladies, with the inflammation and its concomitant phenomena mitigated; and a tendency to assume a milder, but continued, condition of heat, redness, pain, and swelling; and influenced by causes that prevent normal granulation. For example, when from mechanical or chemical causes, a part has been injured and an ulceration established, in a person previously healthy, we get a common or simple ulcer, subject only to such differences as are produced by his temperament, age, occupation, previous habits, surgical dressings, etc. If, now, no untoward constitutional fault exists, with proper local and hygienic measures, we get a healthy sore with nice, clean margins, with numerous small, acuminated, florid, sensitive, and vascular granulations, and a rapid repair of lost parts. Again, take an ulcer of the leg, or, indeed, any other part, and assume that an anæmic condition, or that a scrofulous or other constitutional vice exists, or that a local cause, as a foreign substance or varicose veins, is exerting a deleterious influence, or that the patient has been subjected to injudicious treatment, and we have a chronic or complicated ulcer as the result. When, therefore, a chronic condition has become established in an ulcer, it is always complicated with some other malady. This complication may be *constitutional*, arising from debility or specific diseases; or *local*, resulting from undue inflammation; foreign substances, as pieces of wood, iron, or necrosed bone; or from varicose veins, mechanical influences, or maltreatment.

*Debility* acts by producing an aplastic exudation of lymph; granulations imperfect, few in number, semi-translucent, tall, pale, and apices bulbous; discharge serous; margins indurated and elevated. This is a natural result of defective nutrition. The ulcer in this case becomes chronic, because the lymph will not properly organize, and a healthy action cannot be sustained. This anæmic condition favors a constant serous discharge, and, by this means, a certain amount of nutritive material is continually lost, the waste the ulcer has effected is restored, and still more is required and supplied, until a constitutional habit is established and an issue formed. When this condition is once confirmed, perfect granulation is impossible. If, now, we arrest the discharge with a remedial agent that has a specific action upon the constitution in that direction, (and of these, opium is far the best,) the normal work of granulation will immediately commence, and, in a short time, nature will be found to have completed her work of repair.

Mr. SKEY, of London, in the February No. of the *London Lancet*, 1855, declared that opium, in small and regular doses, would cure a chronic ulcer in an anæmic person. I do not know that he explained the specific operation of this drug in these cases, but no doubt it first partially arrests assimilation and disintegration, and thus diminishes the amount to be excreted through the ulcer. This diminution of the wear and tear of the general system changes the local discharge and modifies the character of the part inflamed, which determines the quality and quantity of lymph secreted and its consequent organization. Let us next consider how the condition of these sores is modified by the action of *specific diseases.* These are characterized by a peculiar, definite, morbid material in the blood, having certain specific effects upon it and its nutritive changes with a tendency to accumulate, and manifesting distinctive local phenomena, each variety electing such tissue or organs as are most naturally its prey, and thus effecting those morbid changes which distinguish the operation of each poison. For example, the specific action of the rheumatic poison is upon the white fibrous tissue; small-pox, measles, and scarlatina are each characterized by a distinct cutaneous disease; syphilis, too, may be said to have its seats of election, though subject to some degree of transformation in its different stages; scrofula, tubercle, and cancer commence, in all probability, in the glandular system, and extend to and destroy or modify neighboring tissue.

The fact is generally admitted, that all foreign substances are, if possible, expelled from the system; therefore, the product of a dyscrasia, or of a diseased organ or tissue, enters at once into the fluid which issues from an ulcer. The result is, that the character of the ulcer is modified by the conjoint causes of the constitutional vice and nature of the fluid issued. Thus, after an ulcer becomes chronic, it assumes precisely the same character, and is prone to the same influences, as the constitution of its subject, and must be treated exactly in accordance with such conditions. But, when the constitution has become habituated to a drainage, and that condition results from a dyscrasia which depends upon a specifically diseased part, except the morbid condition in the latter is overcome, the nutritive interchanges between it and the blood will continue to sustain the dyscrasia; and the arrest of the discharge is a measure which should be wrought with much skill, or the morbid product will accumulate in the blood, and manifest its peculiar characteristics in other situations.

Our considerations then, in diagnosis, should be to ascertain what the complications are; whether merely drainage with debility, or drainage with dyscrasia or specific disease. Each complication is to be met with the most approved treatment, subject to such modifications as the patient's habits, constitution, and local conditions require. Generally, we need not interfere with the discharge; if our patient has proper constitutional treatment, the discharge will need but little attention. Too much haste to dry it up is fraught with danger, it being (if the system is not previously prepared) reäbsorbed and carried into the circulating mass to reäccumulate in some distant part—generally the glandular—and there develope an inflammation, with an aggravation of the dyscrasia.

When there merely exists a sore, subject to drainage and constitutional sympathy, then they can both be suspended until the wound is healed, by opium in sustaining doses, as Mr. SKEY has shown. But Mr. SKEY would find some of his patients laboring under much trouble, if he should treat all ulcers in anæmic persons with opium. Any of the numerous conditions that may be associated with anæmia must invariably be corrected, and then opium will be a safe and sure remedy.

As regards local treatment, but little interference is generally necessary. The points which require especial attention, are:—1. The removal of all foreign substances, as necrosed or carious bone; pieces of wood, iron, and the like; or a tuberculated base, or tissue so diseased as not to be capable of organization, such as are likely to exist in connection with a scrofulous or cachectic diathesis, may be cauterized or excised. 2. To correct acrid excretions which would interfere with the organization of lymph. 3. Protection from air, and the avoidance of the continued application of water, irritating salves, etc. 4. Position and rest.

The following cases may serve to illustrate some of the ideas advanced in the foregoing pages:—

CASE I. Mrs. T., *æt.* 28, had suffered from an ulcer on either leg for six years; during the latter half of this period menstruation had entirely ceased; the discharge from the ulcers increased monthly, and this increase was accompanied by pain and an aggravation of the inflammation; patient was scrofulous, and her system was weakened; prescribed iodide of potassium, alternated with tinct. muriate of iron. No local treatment was adopted, excepting that the ulcers were washed twice a-day with a solution of chlorinated lime and covered with oiled-silk, which was retained with a roller. Rest was also enjoined. At the expiration of twenty days, the treatment was somewhat modified, by the administration of iodide of iron and opium in small doses; gave sitz-bath, with a few daily applications of a solution of sulphate of zinc. This course of treatment, with but slight variation, in thirty-five days reëstablished menstruation, and in sixty days the ulcers were thoroughly healed. Her health remained good, with no tendency to relapse.

CASE II. Mrs. K., *æt.* 41; ulcers on each leg, caused by milk-leg twenty years before. She had employed Dr. W., an irregular practitioner, who dried up the sores by local applications and elevated position. In ten days from the time of her apparent recovery, she was taken very ill. I was summoned, and found her suffering from inflammation of the kidney and bladder. I was not aware of the previous existence of the ulcers, and, not being informed of this fact, I prescribed the usual course of treatment, and after a few days' attendance the patient had apparently recovered. But in about a week, I was again called, and was surprised to find her laboring under a severe attack of hepatitis; prescribed accordingly, and in a short time she was again convalescent. About the time of the recovery from this last attack, a neighbor asked me if "her friend's sickness did not depend upon those fever sores being dried up." I then, for the first time, obtained the full history of the case. I immediately applied blisters to the situation of the ulcers and reëstablished the issue, when her general health was completely restored.

Case III. Miss M., *æt.* 18; ulcer existing on each leg; cause syphilis; menstruation absent for a period of fifteen months; sores appeared twelve months ago; prescribed iodide of potassium in large doses; local application of solution of chloride of lime; air was carefully excluded, and rest of diseased parts enjoined. At the expiration of 45 days, the ulcers were healed, and during that time the menstrual function was restored. At this time, without my knowledge, and contrary to my directions, the iodide was suspended, when the ulcers very soon reäppeared, in connection with an eruption on the face, and the menstrual function was again arrested. The former treatment was resumed, a few days ago, in connexion with the sitz-bath and iron. The patient is still under treatment, with every prospect of again restoring the normal functions.

